# Occupational Transmission of Extensively Drug-Resistant Tuberculosis, France

**DOI:** 10.3201/eid3203.251099

**Published:** 2026-03

**Authors:** Corentin Poignon, Frédéric Vandenbos, Karine Risso, Delphine Viard, Esther Gydé, Alice Gaudart, David Chirio, Nicolas Veziris, Michel Carles

**Affiliations:** Centre d’Immunologie et des Maladies Infectieuses, Sorbonne Université, CNR Mycobactéries, APHP, Paris, France (C. Poignon, E. Gydé, N. Veziris); Hôpital Pasteur Centre de Lutte Antituberculeuse de Nice, Nice, France (F. Vandenbos, K. Risso); Hôpital Archet, Nice (K. Risso, A. Gaudart, D. Chirio, M. Carles); Centre Hospitalier Universitaire de Nice, Nice (D. Viard).

**Keywords:** tuberculosis and other mycobacteria, antimicrobial resistance, respiratory infections, occupational health, extensively drug-resistant tuberculosis, TNF alpha-inhibitors, immunocompromised patient, bacteria, *Mycobacterium tuberculosis*, France

## Abstract

We report occupational transmission of extensively drug-resistant tuberculosis (TB) to a healthcare worker in France receiving tumor necrosis factor α inhibitor therapy. Despite airborne precautions, the healthcare worker contracted TB working in a high-risk unit. This case underscores that immunocompromised healthcare workers should not be assigned to frontline TB care in high-risk settings.

In France, multidrug-resistant tuberculosis (TB) represents <3% of all notified TB cases annually; variable case counts ranged from 44 cases in 2021 to 110 in 2014. The World Health Organization defines extensively drug-resistant TB (XDR TB) by resistance to rifampin, isoniazid, and fluoroquinolones in addition to bedaquiline or linezolid, representing the most difficult-to-treat forms of the disease. Tumor necrosis factor α (TNF-α) inhibitors (e.g., infliximab, adalimumab) disrupt granuloma integrity and increase the risk for latent TB reactivation or new infection by up to 25-fold (*1*). We report a case of occupational transmission of XDR TB to a healthcare worker in France receiving TNF-α inhibitor therapy, despite airborne precautions.

In December 2023, a 57-year-old woman sought care after experiencing several weeks of fatigue. Her medical history included ankylosing spondylitis treated by the TNF-α inhibitor adalimumab since 2017. Pretherapeutic QuantiFERON (QIAGEN, https://www.qiagen.com) testing was negative, and she had no personal history of TB or prior exposure. Since 2020, she had worked as a nursing assistant in an infectious diseases department. 

Computed tomography revealed hypermetabolism of right upper lobe alveolar consolidation (Figure). Because of the absence of sputum production, bronchoscopy with culture was performed. An acid-fast bacilli smear result was negative, but Xpert MTB/RIF Ultra (Cepheid, https://www.cepheid.com) testing detected rifampin-resistant *M. tuberculosis*. Occupational TB transmission was assumed.

**Figure:**
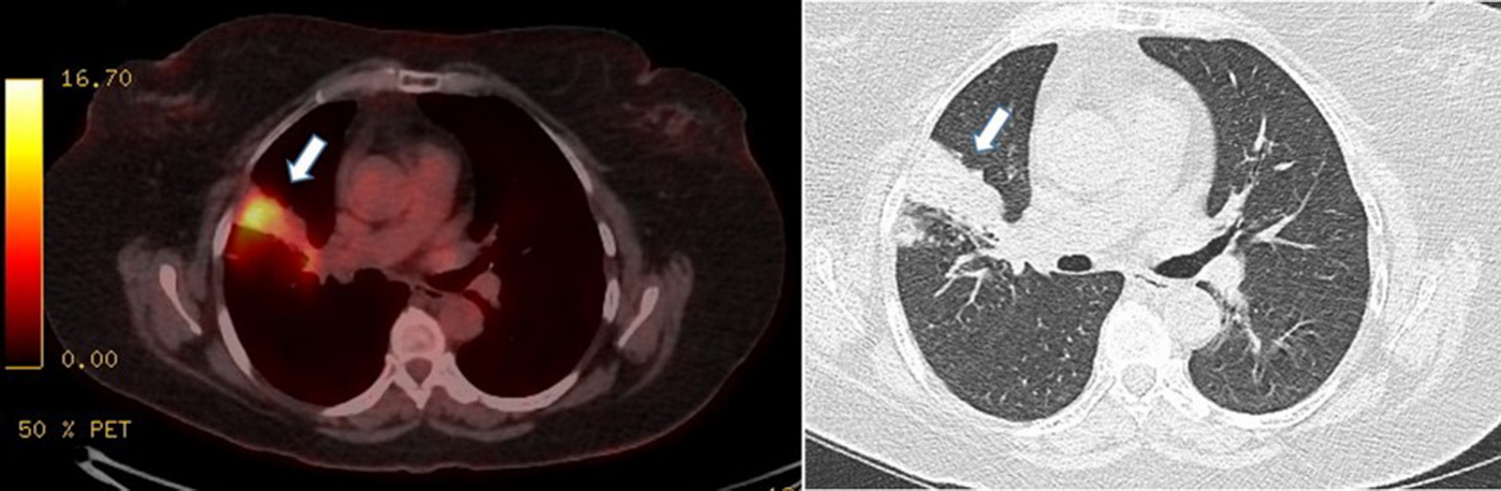
Computed tomography scans of a healthcare workers with occupationally transmitted extensively drug-resistant tuberculosis, France. Scan revealed hypermetabolism (standardized update value 9.1) of the right upper lobe alveolar consolidation, indicated by white arrows.

The bronchoscopy culture was positive and sent to the French National Reference Center for Mycobacteria (Nice, France), where we conducted targeted next-generation sequencing by using Deeplex Myc-TB (Illumina, https://www.illumina.com), whole-genome sequencing analysis (Illumina), and phenotypic drug susceptibility testing. Genotypic analysis revealed resistance to 7 drugs, including rifampin, isoniazid, fluoroquinolones, and an insertion in the *rv0678* gene resulting in a frameshift associated with bedaquiline and clofazimine resistance. Phenotypic susceptibility testing confirmed genotypic resistance to bedaquiline and clofazimine (MICs >2 mg/L) but susceptibility to aminoglycosides, delamanid, linezolid, and cycloserine, confirming XDR TB. 

Before susceptibility test results were available, we started the patient on bedaquiline (400 mg 1×/d for 2 weeks, followed by 200 mg 3×/wk), linezolid (600 mg/d), delamanid (100 mg 2×/d), cycloserine (500 mg/d), and amikacin (10 mg/kg/d) for the first 3 months. Bedaquiline was maintained in the treatment protocol because *rv0678* mutations usually confer low-level MIC increases, and a negative effect on treatment outcome is unconfirmed (*2*). Cultures were identified within 1 month. The patient completed 13 months of treatment (1 year after identification) and remained clinically well with stable imaging at 6 months posttreatment. After a multidisciplinary provider discussion to balance rheumatologic needs and infectious disease risk, TNF-α inhibitor therapy was resumed after 3 months of treatment, clinical improvement, and documented culture identification.

A review of department cases discovered a smear-positive cavitary XDR TB patient hospitalized 3 months earlier. Whole-genome comparison confirmed both isolates were identical (0 single-nucleotide polymorphism difference). The index patient was isolated with airborne precautions, including filtering face piece class 2 mask use by all staff, but he was autonomous, and mask compliance outside direct care activities could not be ensured. He stayed 10 days before he was transferred to another hospital without treatment. During that period, the patient we describe provided routine close-contact care, during which exposure might have occurred.

TNF-α inhibitors have greatly improved outcomes in chronic inflammatory diseases (*1*,*3*). Pretreatment screening for active TB or latent TB is standard practice (*4*,*5*), but infection can still occur after treatment initiation (*4*).

In France, nosocomial TB transmission among healthcare workers has decreased because of declining TB incidence and stringent airborne precautions (*5*). Nevertheless, departments regularly managing TB patients remain high-risk areas (*6*). Among patients receiving TNF-α inhibitors for irritable bowel disease, 44 TB cases were reported, including 6 (14%) in healthcare workers (*7*). Another publication determined that healthcare workers receiving TNF-α inhibitors for irritable bowel disease do not have increased risk for infections except for TB (*8*). Our case confirms this occupational susceptibility in high-exposure environments.

A healthcare worker receiving TNF-α inhibitors who contracted TB despite minimal contact was previously reported (*9*). Genotyping confirmed transmission from a TB patient, suggesting that even brief or indirect exposure might be sufficient for infection under immunosuppressive conditions (*9*). Similar to our case, despite routine infection control measures, exposure to a cavitary XDR TB patient led to transmission. Infection prevention protocols in France are extensive but might not be sufficient for immunosuppressed staff in a TB high-risk unit, especially before efficient treatment is initiated when drug-resistant TB is involved (*10*).

After this event, hospital policy was revised. Newly hired staff already receiving TNF-α therapy will no longer be assigned to pulmonology, infectious diseases, or emergency departments. Current staff in those departments that are beginning TNF-α inhibitors will be offered reassignment. The occupational health revisions reflect a local institutional policy change; further studies will be needed before national or international recommendations can be proposed.

Our case demonstrates confirmed occupational transmission of XDR TB to an immunocompromised healthcare worker despite airborne precautions. Our findings underscore the vulnerability of immunosuppressed healthcare workers and support the need to reconsider their assignment to frontline TB care in high-risk departments.
